# Internal Carotid Artery Stenosis and Collateral Recruitment in Stroke Patients

**DOI:** 10.1007/s00062-017-0568-x

**Published:** 2017-04-24

**Authors:** Jan W. Dankbaar, Kelly G. P. Kerckhoffs, Alexander D. Horsch, Irene C. van der Schaaf, L. Jaap Kappelle, Birgitta K. Velthuis, DUST investigators

**Affiliations:** 1 Department of Radiology, Brain Center Rudolf Magnus, University Medical Center Utrecht, PO-Box 85500, 3508GA Utrecht, The Netherlands; 2 Department of Neurology and Neurosurgery, Brain Center Rudolf Magnus, University Medical Center Utrecht, Utrecht, The Netherlands

**Keywords:** Carotid stenosis, Collaterals, Stroke, CT perfusion

## Abstract

**Purpose:**

Leptomeningeal collaterals improve outcome in stroke patients. There is great individual variability in their extent. Internal carotid artery (ICA) stenosis may lead to more extensive recruitment of leptomeningeal collaterals. The purpose of this study was to evaluate the association of pre-existing ICA stenosis with leptomeningeal collateral filling visualized with computed tomography perfusion (CTP).

**Methods:**

From a prospective acute ischemic stroke cohort, patients were included with an M1 middle cerebral artery (MCA) occlusion and absent ipsilateral, extracranial ICA occlusion. ICA stenosis was determined on admission CT angiography (CTA). Leptomeningeal collaterals were graded as good (>50%) or poor (≤50%) collateral filling in the affected MCA territory on CTP-derived vessel images of the admission scan. The association between ipsilateral ICA stenosis ≥70% and extent of collateral filling was analyzed using logistic regression. In a multivariable analysis the odds ratio (OR) of ICA stenosis ≥70% was adjusted for complete circle of Willis, gender and age.

**Results:**

We included 188 patients in our analyses, 50 (26.6%) patients were classified as having poor collateral filling and 138 (73.4%) as good. Of the patients 4 with poor collateral filling had an ICA stenosis ≥70% and 14 with good collateral filling. Unadjusted and adjusted ORs of ICA stenosis ≥70% for good collateral filling were 1.30 (0.41–4.15) and 2.67 (0.81–8.77), respectively. Patients with poor collateral filling had a significantly worse outcome (90-day modified Rankin scale 3–6; 80% versus 52%, *p* = 0.001).

**Conclusion:**

No association was found between pre-existing ICA stenosis and extent of CTP derived collateral filling in patients with an M1 occlusion.

## Introduction

It is well known in acute ischemic stroke patients that good collateral blood supply to the area behind an arterial occlusion is an important determinant of clinical recovery and favourable outcome [1–8]. This collateral circulation can be divided into primary and secondary collaterals. The primary collaterals include the arterial segments of the circle of Willis. The secondary collaterals consist of the ophthalmic arteries and leptomeningeal vessels [9]. Interestingly, there is great variability in leptomeningeal collateral circulation in stroke patients [10]. The clinical and demographic variables that influence the degree of collateral circulation are inconsistent and poorly understood [11]. Collateral circulation is known to develop in peripheral and coronary arterial steno-occlusive disease [12]. It has also been hypothesized that chronic internal carotid artery (ICA) stenosis may lead to a more profound recruitment of collateral circulation [9, 13]. Direct visualization of collateral filling can be achieved with angiographic methods, such as computed tomography angiography (CTA), magnetic resonance angiography (MRA) and conventional angiography. Leptomeningeal collaterals can be accurately visualized by CTA due to a high spatial resolution [14]; however, the assessment of collaterals is dependent on the timing of image acquisition. As collaterals are often enhanced later than the primary circulation, they are visualized better with CT perfusion (CTP) derived vessel imaging than with single-phase CTA [15–17].

The purpose of this study was to investigate the relationship between a pre-existing stenosis of the ICA and the recruitment of leptomeningeal collaterals visualized with CTP.

## Methods

### Study Design

All patients participated in a prospective, multicenter, observational, acute stroke study in which the prognostic value of CT within 9 h after onset of symptoms of acute ischemic stroke was evaluated [18]. All procedures performed in this study were in accordance with the ethical standards of the institutional medical ethics committee. All patients or family gave signed informed consent, unless a patient died before consent could be obtained, in that case the need for consent was waived by the medical ethics committee.

### Patient Selection

All patients with an occluded M1 segment (with or without extension in the intracranial ICA or M2 branches) on admission CTA were retrospectively selected. Patients with only distal occlusions (>M1) were excluded, since in these patients it cannot be accurately determined to what extent the affected part of the MCA flow territory receives blood supply from leptomeningeal collaterals. Patients with ipsilateral and extracranial ICA occlusions were excluded, because the presence of pre-existent ICA stenosis cannot be determined in these patients. Patients with a posterior circulation stroke were excluded since the focus of this study was on MCA territory collateral filling.

### Patient Characteristics

For all patients demographic data were obtained on age, sex, admission National Institutes of Health stroke scale (NIHSS), history of hypertension, diabetes, hyperlipidemia, and current smoking. The stroke subtype was determined using the TOAST classification [19]. The functional outcome after 90 days was determined using the modified Rankin scale (mRs). Poor functional outcome was defined as an mRs of 3–6 and favorable outcome as an mRs of 0–2.

### Imaging Protocol

The protocols for CTA and CTP imaging have been reported previously [18, 20].

All CTA and CTP imaging were performed with 40–320 detector CT scanners (Philips, Best, the Netherlands; Siemens, Erlangen, Germany; GE, Little Chalfont, United Kingdom; Toshiba, Otawara-shi, Japan) covering at least both ASPECTS levels. The CTP scans were performed with 80 kV, 150 mA, and 0.625 mm slice thickness: 40 ml of non-ionic contrast material followed by 40 ml of saline was injected with a flow of 6 ml/s. Images were acquired every 2 s for 50 s after the initiation of contrast injection. The thin slice acquisition is directly reconstructed to 5 mm slice thickness on the CT scanner with no overlap. Using commercially available post-processing software (Extended Brilliance Workstation version 4.5, Philips Healthcare), temporal maximum intensity projections (tMIP) of the 5 mm slices were made by detecting the pixel with the highest attenuation across all time frames on each slice. This enhances the visibility of all vessels within the acquired time frames and results in images similar to CTA, only timing-independent [16]. The CTA was acquired from the aortic arch to the cranium vertex using 50–70 ml of contrast material followed by 40 ml of saline with a flow of 6 ml/s.

### Imaging Evaluation

**Fig. 1:**
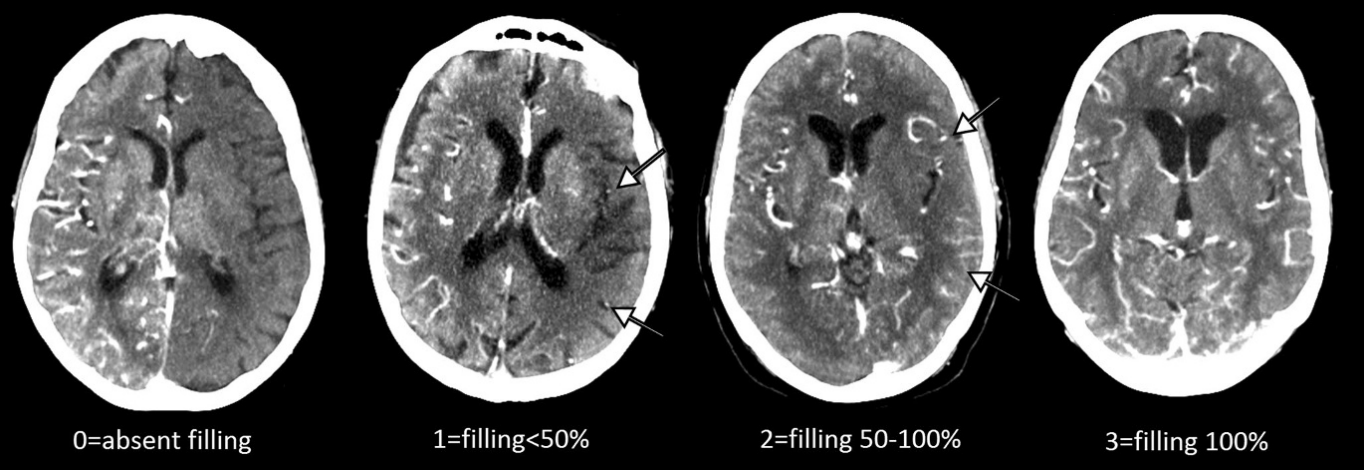
Examples of the different grades of collateral filling as seen on temporal maximum intensity projections (tMIP) derived from 5 mm CTP slices by detecting the pixel with the highest attenuation across all time frames on each slice. *Arrows* indicate the filling vessels in grade 1(≤50%) and 2 (>50 but <100%) [4]

Stenoses of the ICA and ECA were determined on CTA using NASCET ratios [21]. Stenosis grade on CTA correlates well with the gold standard DSA [22]. The circle of Willis was evaluated on CTA and considered complete when all routes to the occluded M1 (Acom and bilateral A1, ipsilateral Pcom and P1) showed contrast-enhanced arteries with a diameter ≥1 mm. All CTA images were evaluated by one of three experienced observers. Leptomeningeal collaterals were graded on admission CTP derived tMIP by the extent of collateral filling in the affected hemisphere: 0 = absent; 1 = filling ≤ 50%; 2 = filling > 50–<100%; 3 = filling 100% (Fig. 1; [4]). For the analyses scores were dichotomized into poor collateral filling (0–1) and good collateral filling (2–3).

All CTP images were evaluated by two observers in consensus, blinded to clinical information at the time of evaluation, except for the side of the occlusion.

### Statistical Analysis

Statistical analyses were performed using SPSS 20.0 (IBM Corporation, Armonk, NY). Patient characteristics and imaging findings were compared between patients with good and poor collaterals using the Mann-Whitney U‑test for continuous variables and a Pearson χ^2^-test for categorical variables. Logistic regression analysis was used to calculate the unadjusted and adjusted odds ratio (OR) and corresponding confidence interval of ICA stenosis ≥70% for good collaterals. The OR was adjusted for age, gender, and complete circle of Willis. A complete circle of Willis is a possible confounder in the relationship between ICA stenosis and collateral grade. In a sub-analysis the unadjusted OR of ICA stenosis ≥50% for good collaterals was calculated.

## Results

We identified 209 patients with an occluded M1 segment but without ipsilateral, extracranial ICA occlusion. Of the patients 21 were excluded: 14 patients with poor image quality and 7 with a scanning level that was too low (at the level of the posterior circulation) to evaluate collateral filling in the MCA territory. Of the remaining 188 patients 50 (26.6%) were classified as having poor collateral filling and 138 (73.4%) as good collateral filling. Patient characteristics are summarized in Table 1. Admission NIHSS was significantly lower in patients with good collateral filing. Patients with poor collateral filling had a significantly worse outcome (90-day mRs 3–6) than patients with good collateral filling (80% versus 52%, *p* = 0.001).

**Table 1:** Patient characteristics

	(*N* = 188)	Good collaterals (*N =* 138)	Poor collaterals (*N =* 50)	*p*-value
Age, years, median (IQR)	68.3 (55.8–77.1)	68.7 (54.0–76.9)	67.5 (56.2–78.7)	0.688
Sex, male, *N* (%)	101(53.7)	72(52.2)	29(58.0)	0.511
NIHSS, median (IQR)	15(9–19)	13(9–17)	19(14–22)	**<0.001**
History of, *N* (%):				
– Hypertension	94(50.0)	67(48.6)	27(54.0)	0.621
– Diabetes	22(11.7)	16(11.6)	6(12.0)	1.000
– Hyperlipidemia	51(27.1)	33(23.9)	18(36.0)	0.142
– Smoking	98(52.1)	75(54.3)	23(46.0)	1.000
ICA stenosis ≥70%, *N* (%)	18(9.6)	14(10.1)	4(8.0)	0.785
CoW complete, *N* (%)	65(34.6)	46(33.3)	19(38.0)	0.604
90-day mRs 3–6, *N* (%)	113(60%)	73(52%)	40(80%)	**0.001**

*IQR* interquartile range, *NIHSS* National Institute of Health stroke scale, *CoW* circle of Willis, *ECA* external carotid artery, *ICA* internal carotid artery, *mRs* modified Rankin scale

Of the patients 18 had an ICA stenosis ≥70% ipsilateral to the M1 occlusion, 15 of the 18 patients were diagnosed with large vessel atherosclerosis, 1 with cardioembolism and 2 with another determined etiology using the TOAST classification. Of the with good collateral filling 14 (10.1%) patients had an ICA stenosis ≥70% and 4 (8%) with poor collateral filling. The unadjusted and adjusted OR and corresponding 95% confidence interval (CI) of ICA stenosis ≥70% (*n* = 18) for good collateral filling was 1.30 (0.41–4.15) and 2.67 (0.81–8.77), respectively. For ICA stenosis ≥50% (*n* = 23) the unadjusted OR for good collateral filling was 1.35 (0.47–3.85).

## Discussion

No relationship was found between a pre-existing ipsilateral ICA stenosis and the extent of leptomeningeal collateral supply on CTP in patients with acute ischemic stroke from an M1 occlusion. Our results are in contrast with a previous study showing a significant relationship between the severity of ICA stenosis and collateral filling using digital subtraction angiography (DSA) [23]. However, no difference was made between primary and secondary collateral pathways in this study. In addition, patients without a cerebral artery occlusion and patients with anterior cerebral artery and posterior circulation occlusions were also included. A different study, looking at collateral recruitment and ICA occlusion, found no association between contralateral ICA stenosis and secondary collateral supply using DSA [24]. Our study is essentially different from other studies since we only studied ipsilateral ICA stenosis and collateral filling in patients with an occluded M1 MCA segment. In our opinion, the absence of a proximal intracranial occlusion makes the evaluation of the extent of collateral recruitment challenging if not impossible, since the primary supply route is still patent. Although DSA is the reference standard for imaging collaterals, most hospitals use CT for the initial work-up of acute ischemic stroke patients [25]. On single-phase CTA, the assessment of collateral filling depends largely on the timing of imaging, and delayed contrast enhancement of collateral vessels can lead to an underestimation of collateral blood supply [15, 26]. All vessels, including collateral pathways with delayed filling, are visualized on a CTP derived tMIP.

There are clear differences between collateral grading systems. In a recent review, seven systems for classifying collaterals on CTA images were identified [27]. Interobserver correlation was assessed for five of them, ranging from low to excellent (kappa = 0.494–0.93). The system used in the current study had an interobserver correlation of 0.87 (Tan system [4]). The Tan system was designed for CTA and has not yet been validated for CTP; however, CTP derived tMIP images result in similar images of blood vessels as CTA, only timing-independent.

Unlike our study, a smaller study (*n* = 54) using CTP derived collateral filling, with the same collateral grading system found an association between poor collateral filling and diabetes but not with initial stroke severity [6]. As in our study it was shown that collateral filling predicts early neurologic recovery. Two other studies have also shown that CTP derived collateral filling predicts functional outcome in acute stroke patients [15, 16]. One of these studies showed that the association was highest when using CTP derived tMIP images [16].

Our study has some limitations. First, we could not compare our results with the reference standard conventional angiography. Second, although we evaluated a moderate sized cohort of 188 patients, only 18 patients had an ICA stenosis ≥70%. Third, we only determined the ICA stenosis grade at the time of admission to the emergency department. No information was available regarding the chronicity of the detected stenosis. It can therefore not be excluded that the stenosis only developed recently and consequently had not yet resulted in leptomeningeal collateral recruitment. Moreover, it has been hypothesized that leptomeningeal collaterals may rapidly open up once a pressure gradient passes a certain threshold due to an acute occlusion [28]. Fourth, the tMIP images used in this study were obtained from 5 mm slices. This results in less vessel detail compared to MIP images reconstructed from thin 0.625 mm slices. Unfortunately, thin slice reconstructions were not available in all patients. Although this may be an issue if small vessel occlusions need to be identified [26], this will most likely not influence the grade of collateral filling since it is based on regional visibility of vessels. The visualization of delayed filling and not the visibility of small vessels was deemed to be the most important aspect of the use of CTP in this study [15].

In conclusion, no association was found between a pre-existing ipsilateral ICA stenosis and the extent of leptomeningeal collaterals assessed with CTP in acute ischemic stroke patients with an M1 occlusion. Therefore, this study did not find support for the hypothesis that ICA stenosis leads to more recruitment of leptomeningeal collaterals; however, the low number of patients with a proximal stenosis may have influenced these results.
